# A Review on the Association between Glucagon-Like Peptide-1 Receptor Agonists and Thyroid Cancer

**DOI:** 10.1155/2012/924168

**Published:** 2012-05-28

**Authors:** Wei-Yih Chiu, Shyang-Rong Shih, Chin-Hsiao Tseng

**Affiliations:** ^1^Department of Internal Medicine, National Taiwan University College of Medicine, Taipei 10002, Taiwan; ^2^Division of Endocrinology and Metabolism, Department of Internal Medicine, National Taiwan University Hospital, Taipei 10002, Taiwan

## Abstract

There is a concern on the risk of thyroid cancer associated with glucagon-like peptide-1 (GLP-1) analogs including liraglutide and exenatide. In this article, we review related experimental studies, clinical trials and observational human studies currently available. In rodents, liraglutide activated the GLP-1 receptors on C-cells, causing an increased incidence of C-cell neoplasia. Animal experiments with monkeys demonstrated no increase in calcitonin release and no C-cell proliferation after long-term liraglutide administration. Longitudinal 2-year data from clinical trials do not support any significant risk for the activation or growth of C-cell cancer in humans in response to liraglutide. However, an analysis of the FDA adverse event reporting system database suggested an increased risk for thyroid cancer associated with exenatide after its marketing. Noticeably, a recent study discovered that GLP-1 receptor could also be expressed in human papillary thyroid carcinomas (PTC), but the impact of GLP-1 analogs on PTC is not known. Therefore, GLP-1 analogs might increase the risk of thyroid C-cell pathology in rodents, but its risk in humans awaits confirmation. Since GLP-1 receptor is also expressed in PTC besides C-cells, it is important to investigate the actions of GLP-1 on different subtypes of thyroid cancer in the future.

## 1. Introduction

Glucagon-like peptide-1 (GLP-1) is an incretin hormone released after meals by L cells in the ileum [1]. It increases the secretion of insulin from the pancreas in a glucose-dependent manner and suppresses the secretion of glucagon, a counter-hormone to insulin [2]. There are two GLP-1-mimetic drugs currently approved for clinical use to treat type-2 diabetes, that is, exenatide and liraglutide [3, 4]. Exenatide is the first GLP-1 receptor agonist approved in April 2005 for the treatment of type-2 diabetes mellitus. It is a 39-amino acid peptide with 53% amino acid homology to full-length GLP-1 [4]. With elimination by glomerular filtration [5] and a mean half-life of 3.3–4 hours [6], exenatide has to be injected subcutaneous twice a day. On January 25, 2010, the FDA approved liraglutide, a GLP-1 receptor agonist that can be injected once daily to improve glycemic control in adults with type-2 diabetes [3, 4]. Liraglutide is a long-acting GLP-1 analog with one amino acid substitution (Arg34Lys) and an attachment of a C-16-free-fatty acid derivative via a glutamol spacer to Lys26 [4]. These modifications lead to slower absorption rate from injection site, higher binding affinity to albumin, and a plasma half-life of 11–13 hours [7–9]. While GLP-1 analogs can efficiently reduce blood glucose level in patients with type-2 diabetes [3, 4], they may potentially have adverse effects on thyroid glands because GLP-1 receptors are expressed in thyroid glands of humans [10] as well as in those of rodents [11]. In preclinical animal studies, rodents treated with liraglutide would have a higher incidence of C-cell tumor formation and focal hyperplasia [12, 13]. It is possible that long-term exposure to GLP-1 receptor agonists in humans may also induce C-cell neoplasia since GLP-1 receptors are expressed in the human thyroid glands [10].

Both the prevalence and incidence of diabetes have been increasing dramatically in recent decades, especially in the Asian people [14]. Diabetes is also one of the leading causes of death nowadays [15]. The link between diabetes and cancers has become a great concern recently, and the use of antidiabetic drugs may partially contribute to such an increased cancer risk in the diabetic patients [16–25]. For examples some clinical trials have suggested an association between pioglitazone and bladder cancer [26, 27]. In this paper, we reviewed experimental studies, controlled clinical trials, and observational human studies currently available on the association between GLP-1 analogs and thyroid cancer.

## 2. Experimental *In Vitro* and Animal Studies in Rodents

Calcitonin, a hormone secreted by thyroid C cells, is regarded as an important clinical biomarker for C-cell diseases such as medullary thyroid carcinoma (MTC) and hereditary C-cell hyperplasia because of its high sensitivity and specificity [28–30]. Several *in vitro* studies employing rat thyroid C-cell lines and thyroid tissues have demonstrated that activation of the GLP-1 receptor leads to calcitonin secretion, which is attenuated by the GLP-1 receptor antagonist exendin (9–39) [31, 32]. The functional effect of GLP-1 receptor agonists on rat C-cell lines was investigated by Knudsen et al. [11]. They found that GLP-1 receptor agonists elicited calcitonin release and calcitonin gene expression in a dose-dependent manner in rodent C cells. GLP-1 receptor agonists, including native GLP-1, exenatide, and liraglutide, activated rodent thyroid C cells to release calcitonin in a GLP-1 receptor-dependent manner.

In addition to the *in vitro* studies, Knudsen et al. designed animal experiments to study the development of C-cell hyperplasia and tumor formation after long-term dosing with GLP-1 receptor agonists in rodents [11]. The incidences of both C-cell hyperplasia and C-cell tumor formation at 104 weeks increased in a dose-dependent manner and reached statistical significance. The minimum doses of liraglutide to cause a statistically significant increase in the incidence of C-cell hyperplasia were 0.25 mg/kg/day in rats and 0.2 mg/kg/day in mice. Both doses were 2-fold greater than the equivalent human dose of 1.8 mg/day [11, 12]. The minimum doses to induce a significant increase in C-cell tumor formation in rats and mice were 0.075 mg/kg/day and 1.0 mg/kg/day, respectively; the dose in rats (0.075 mg/kg/day) was equivalent to the dose recommended for treatment of type-2 diabetes in humans [11, 12]. On the other hand, C-cell tumors occurred in mice receiving a daily dose of liraglutide that was 10-fold greater than the corresponding human dose [11, 12]. Of note, in 2-year studies involving wild-type mice, dose-dependent C-cell hyperplasia and neoplasia developed only at doses that also caused increased calcitonin levels [11]. Together, *in vitro* and *in vivo* experiments have demonstrated that long-term GLP-1 receptor activation is associated with increased calcitonin gene transcription and subsequently with C-cell proliferation and tumor formation in both rats and mice.

Madsen et al. [33] documented that C-cell hyperplasia and calcitonin release associated with GLP-1 agonists in wild-type mice were GLP-1-receptor dependent. Besides, C-cell effects seen in mice were not associated with the activation of the rearranged-during-transfection (RET) protooncogene. GLP-1 activates the mammalian target of rapamycin (mTOR) pathway by stimulating the production of cAMP. Activation of mTOR in turn results in downstream phosphorylation of ribosome S6. In contrast, these effects were not observed in GLP-1-receptor knock-out mice.

## 3. Experimental *In Vitro* and Animal Studies in Primates

In addition to their experiments in rodents, Knudsen et al. [11] investigated the effect of GLP-1 receptor agonists on human TT C cells and on nonhuman primates (cynomolgus monkeys). In contrast to the results in rodents, GLP-1 receptor agonists did not stimulate calcitonin release in human TT C cells. The expression of GLP-1 receptor in human TT C cells was very low, and the corresponding mRNA transcripts were 14- to 21-fold lower than in rat C cells. Furthermore, *in vivo* animal studies with cynomolgus monkeys demonstrated no calcitonin release and no effects on the relative C-cell fraction in the thyroid gland or C-cell proliferation in monkeys after up to 87 weeks dosing with liraglutide at 5.0 mg/kg/day. This liraglutide dose was up to 60-fold greater than the highest dose recommended for the treatment of type-2 diabetes.

## 4. Human Clinical Trials and Epidemiologic Studies

Additionally, a series of clinical trials in over 5,000 patients with either diabetes or nondiabetic obesity was presented by Hegedüs et al. [34]. In all trials, subjects were randomized to receive liraglutide at doses ranging from 0.6 to 3.0 mg, active comparator, and/or placebo. Calcitonin concentration was monitored at baseline and at 12-week intervals, thereafter, in all subjects enrolled in 8 phase-3 clinical trials (the liraglutide effect and action in diabetes (LEAD) trials 1–6, and 2 phase-3 trials in Japanese subjects) and in 1 phase-2 trial with nondiabetic obese subjects. These trials had intervention phases of 20 to 104 weeks. When combining data from these 9 clinical studies, there was no consistent change in calcitonin levels over time (up to 104 weeks) with any dose of liraglutide or between treatment groups in the proportion of patients whose calcitonin levels increased above a clinically relevant cut-off value of 20 pg/mL. The proportion of subjects switching to a higher calcitonin category, defined by the upper normal range (UNR; <UNR, UNR-2UNR, ≥2UNR) or by potential calcitonin signals of 20, 50, and 100 ng/L for C-cell abnormalities (<20 ng/L, 20 to <50 ng/L, 50 to <100 ng/L, and ≥100 ng/L), was low and did not differ between the liraglutide and active comparator groups. In the LEAD-6 trial [3], the calcitonin responses to distinct GLP-1 receptor agonists (liragutide 1.8 mg once daily versus exenatide 10 *μ*g twice daily) were compared. No difference was seen in estimated geometric mean calcitonin over 26 weeks between the 2 groups. In the trial with nondiabetic obese subjects, treatment groups receiving a higher liraglutide dose (1.2–3 mg/day) were compared with those receiving orlistat (360 mg/day) or placebo. The estimated geometric mean calcitonin levels over 52 weeks remained at the low end of the normal range in all treatment groups. Regarding clinical adverse events related to C cells, 6 subjects were found to have histologically documented C-cell hyperplasia with identical prevalence in the liraglutide and nonliraglutide groups. One case of MTC was described in a subject who was not treated with liraglutide. Although there were fluctuations in calcitonin levels among these 7 subjects during the trial periods, no consistent pattern was discovered. Taking together, these data do not support any significant risk for the activation or growth of C cells in humans in response to GLP-1 receptor agonists over the 2-year period.

Although GLP-1 receptor stimulation induced calcitonin release and C-cell proliferation in rodents, these effects were not observed in primates [11], implying possible species-specific differences in GLP-1 receptor expression and activation in the thyroid. Computer-assisted cell counting in sections stained immunohistochemically for calcitonin revealed that the C-cell densities in thyroid glands from cynomolgus monkeys and humans were comparable, and more importantly, that the C-cell densities in thyroid glands in mice and rats were 22- and 45-fold higher, respectively, than that in humans [11].

## 5. GLP-1 Receptor Expression in Thyroid Tumors Derived from Follicular Cells and Its Functional Significance

Recently, Gier et al. [10] examined thyroid tissue samples procured at surgery from individuals with C-cell hyperplasia and those with MTC for the presence of GLP-1 receptor expression using immunocytochemical techniques. C-cells within relatively normal tissue without any hyperplasia or neoplastic changes were also evaluated for GLP-1 receptor expression. In this study, calcitonin-expressing C cells were immunoreactive for GLP-1 receptor in 33% (5/15), 91% (10/11), and 100% (9/9) of individuals with normal thyroid lobes, MTC, and C-cell hyperplasia, respectively. There was no immunoreactivity for either calcitonin or the GLP-1 receptor in normal thyroid follicles identified in the same sections. Furthermore, there was no correlation between the extent of GLP-1 receptor immunoreactivity and either tumor size or plasma calcitonin concentrations. Moreover, the expression of calcitonin and GLP-1 receptors was investigated in thyroid tissues obtained from 17 individuals with papillary thyroid cancer (PTC). PTC cells were negative for calcitonin. But GLP-1 receptor immunoreactivity was unexpectedly present in PTC cells in 3 of the 17 (18%) cases. In other words, the GLP-1 receptor is not expressed in normal thyroid follicular cells but may be aberrantly present in a subset of PTCs derived from follicular cells. The functional significance of these findings has not yet been ascertained. PTC is the most common malignancy of the thyroid, constituting approximately 50–90% of thyroid malignancies worldwide [35]. Thus, GLP-1 receptor immunopositivity in a subset of PTC lesions is likely to be of greater epidemiological significance than in C-cell neoplasms. Elashoff et al. [36] examined the FDA adverse event reporting system (AERS) database for thyroid cancer in association with exenatide, another human GLP-1 analog. The reported event rate for thyroid cancer was 4.73-fold greater in patients treated with exenatide compared to the control drug, rosiglitazone (*P* = 4 × 10^−3^). Although it is impossible to know the thyroid cancer subtypes reported in FDA AERS database, these findings indicate that it is important to carefully monitor individuals exposed to long-term pharmacological GLP-1 analogs for any increased incidence of thyroid malignancies. In addition, more detailed future investigation into the actions of GLP-1 on each subtype of thyroid cancers, especially PTC, is required.

## 6. Conclusion

Table 1 summarizes the findings on thyroid cancers after dosing with GLP-1 analogs from experimental studies, controlled clinical trials and observational studies (Table 1). Data from studies in rodents suggested that GLP-1 analogs were associated with an increased risk of thyroid C-cell hyperplasia and C-cell tumors. On the other hand, animal experiments with monkeys did not show increased calcitonin release or proliferation of C-cells in thyroid glands after chronic administration of liraglutide. Longitudinal data from clinical trials have not demonstrated a causal association between GLP-1 analogs and thyroid C-cell pathology over a 2-year period. However, long-term observational studies are required to monitor such a potential risk in human. It is worth noting that a subset of human PTC expresses GLP-1 receptors. The FDA AERS database supported an increased risk of thyroid cancer associated with exenatide. It is urgent to investigate the actions of GLP-1 on each subtype of thyroid cancers, especially PTC, in the future. Besides, caution is warranted in the use of this class of agents, especially in individuals with a history of thyroid cancer.

## Figures and Tables

**Table 1:** *In vitro*/*in vivo* experiments and clinical studies on the association between GLP-1 analogs and C-cell pathology.

Authors [reference]	Studied drugs	Research materials or study subjects	Main outcomes investigated	Main findings
			Cellular models	

Crespel et al. [32]	Glucagon, GLP-1 (7–36), and exendin (9–39)	Rat CA-77 C-cell line	cAMP production and calcitonin secretion	GLP-1 (7–36) and glucagon dose dependently stimulated cAMP production and calcitonin secretion. Exendin (9–39) abolished a further increase in cAMP formation at glucagon concentration over 10 nM and partially suppressed glucagon-induced calcitonin secretion.

Lamari et al. [31]	GLP-1 (7–37), and exendin (9–39)	Rat CA-77 C-cell line	cAMP production, calcitonin mRNA levels, and calcitonin secretion	GLP-1 (7–37) increased cAMP formation in a dose-dependent manner. Exedin (9–39), an antagonist of GLP-1 receptor, blunted the stimulation of cAMP production induced by GLP-1 (7–37). Gene expression and peptide secretion of calcitonin were increased after incubation of CA-77 cells with GLP-1 (7–37).

Knudsen et al. [11]	Liraglutide, exenatide, and GLP-1 (7–37)	Human TT C-cell line, rat MTC 6–23 C-cell line, and rat CA-77 C-cell line	GLP-1 receptor mRNA and protein expression; calcitonin release after GLP-1 receptor agonists	Native GLP-1, liraglutide, and exenatide all stimulate calcitonin gene expression and calcitonin secretion via the GLP-1 receptor in a dose-dependent manner in rat C cells. The human TT cells express few GLP-1 receptors compared with rat MTC 6–23 and CA-77 and show a lack of functional response to GLP-1 and GLP-1 receptor agonists.

			Animal experiments	

(I) Rodents				
Knudsen et al. [11]	Liraglutide versus vehicle control	Sprague Dawley rats aged 6-7 weeks and CD-1 mice at the age of 5–10 weeks	Plasma calcitonin and pathological examination to thyroid gland sections after dosing with liraglutide	Calcitonin levels increase with time and dose with 104-week repeated dosing of liraglutide. The incidences of both C-cell hyperplasia and C-cell tumor formation at 104 weeks were increased in a dose-dependent manner and reached statistical significance.

Madsen et al. [33]	Liraglutide, exenatide, and vehicle control	CD-1 wild-type mice aged 5-6 weeks and GLP-1-receptor knockout mice at the age of 4-5 weeks	Plasma calcitonin, pathological examination to thyroid tissue sections, and immunohistochemical staining for phosphoproteins after 13-week treatment with liraglutide or exenatide	GLP-1 agonists cause calcitonin release and C-cell hyperplasia in wild-type mice via a GLP-1-receptor-dependent mechanism. GLP-1 activates the mammalian target of rapamycin (mTOR) pathway by stimulating the production of cAMP. Activation of mTOR in turn results in downstream phosphorylation of ribosome S6. Liraglutide-induced C-cell hyperplasia in mice is not associated with RET activation.

(II) Primates				
Knudsen et al. [11]	Liraglutide versus vehicle control	Cynomolgus monkeys aged 1-2 years	Plasma calcitonin and pathological analysis to thyroid gland sections after dosing with liraglutide	No increase in plasma calcitonin was seen in cynomolgus monkeys receiving a single dose of liraglutide or during 87-week daily dose. There was also no change in the thyroid gland sections, relative C-cell fraction of the thyroid gland, and proliferative index in the C cells after liraglutide for 52 weeks.

			Human studies	

Gier et al. [10]	—	Human thyroid glands	Expression of GLP-1 receptors in tissue samples with C-cell abnormalities, papillary thyroid cancer, and normal thyroid	GLP-1 receptor immunoreactivities were detected in 33%, >90%, and 18% of patients with normal C cells, C-cell pathologies, and PTC lesions, respectively.

Hegedüs et al. [34]	Liraglutide versus active comparators and placebo	Nine clinical trials of 20–104-week duration	Geometric mean levels of serum calcitonin and outlier analysis	There was no significant difference in mean calcitonin levels between liraglutide and control groups. The proportions of subjects with calcitonin levels shifting to a higher category or above a clinically relevant cut-off value of 20 pg/mL were low and did not differ between treatment groups.

Elashoff et al. [36]	Exenatide versus rosiglitazone	Adverse effect reporting system	Overall thyroid cancer	Odds ratio for thyroid cancer was 4.73; *P* = 4 × 10^−3^
